# Thymic stromal lymphopoietin promotes abdominal aortic aneurysm formation by regulating macrophage polarization

**DOI:** 10.3389/fimmu.2026.1767913

**Published:** 2026-04-07

**Authors:** Qi Zheng, Xuhong Wang, Xinglin Chen, Mingkai Huang, Xuzhe Zhang, Qinlin Li, Zhengjie Huang, Mingyi Li, Jingyong Li, Wangling Hu, Ni Xia, Lingfeng Zha, Jiao Jiao, Fen Yang, Tingting Tang, Xiang Cheng

**Affiliations:** 1 Department of Cardiology, Union Hospital, Tongji Medical College, Huazhong University of Science and Technology, Wuhan, China; 2 Hubei Key Laboratory of Biological Targeted Therapy, Union Hospital, Tongji Medical College, Huazhong University of Science and Technology, Wuhan, China; 3 Hubei Provincial Engineering Research Center of Immunological Diagnosis and Therapy for Cardiovascular Diseases, Union Hospital, Tongji Medical College, Huazhong University of Science and Technology, Wuhan, China

**Keywords:** abdominal aortic aneurysm, chemokine signaling pathway, inflammation, macrophage polarization, thymic stromal lymphopoietin

## Abstract

**Background:**

The inflammatory cytokine thymic stromal lymphopoietin (TSLP) has pleiotropic roles in immune response and tissue homeostasis, yet its function in abdominal aortic aneurysm (AAA) remains entirely unexplored.

**Methods:**

We integrated clinical epidemiology, *in vivo* experimental models, and *in vitro* mechanistic assays. Using the UK Biobank (UKB), we analyzed serum TSLP levels in 632 AAA patients and 24,036 controls. Using two murine AAA models (porcine pancreatic elastase and calcium phosphate), function of TSLP was evaluated by genetic deletion of Tslp receptor (Tslpr), administration of a neutralizing anti-TSLP monoclonal antibody, and exogenous TSLP delivery. The immune landscape of aorta was profiled by mass cytometry (CyTOF). The effects of TSLP on macrophage polarization was assessed *in vitro*. Underlying mechanisms were probed by bulk RNA sequencing.

**Results:**

AAA patients showed a markedly elevated serum TSLP. In murine models, TSLP was increased in the aortic tissues, primarily derived from fibroblasts. Genetic ablation of Tslpr and pharmacological neutralization of TSLP attenuated the severity of AAA, while exogenous TSLP exacerbated the disease. CyTOF analysis revealed that TSLP signaling skewed the aortic macrophage balance toward the pro-inflammatory M1 phenotype rather than the anti-inflammatory M2 phenotype. *In vitro*, TSLP directly induced M1 while inhibited M2 macrophage polarization. Transcriptomics identified that TSLP upregulated pro-inflammatory pathways in macrophages, including positive regulation of cytokine production, regulation of immune effector process, cytokine−cytokine receptor interaction and chemokine signaling pathway.

**Conclusions:**

Our integrated multi-level analysis has identified TSLP as a novel pathogenic driver of AAA, which promotes disease progression by reprogramming macrophage polarization. These findings nominate TSLP signaling as a mechanistically grounded and therapeutically promising target for AAA.

## 1 Introduction

Abdominal aortic aneurysm (AAA) involves a permanent and localized dilation of the infrarenal aorta, resulting from progressive weakening of the vessel wall. It frequently remains asymptomatic or presents with nonspecific abdominal discomfort until rupturing, a catastrophic complication associated with high mortality (1). In developed countries, AAA affects approximately 1.2% to 3.3% of the elderly population, with a pronounced male predominance (2, 3). For patients with large (diameter >55 mm), symptomatic or ruptured aneurysms, endovascular repair or open surgery remains the standard of care (4). However, no effective pharmacotherapies are currently available to halt the progression of small, asymptomatic AAAs, highlighting a critical unmet clinical need.

The pathogenesis of AAA involves a cascade of pathological alterations, including proteolytic degradation of the extracellular matrix, apoptosis of vascular smooth muscle cells, immune cell infiltration, and oxidative stress within the aortic wall (5). These processes collectively lead to progressive wall weakening and dilation (6). Among these, chronic inflammation driven by innate and adaptive immune responses is now recognized as a central driver of AAA progression (7). In particular, macrophages play a pivotal role through pro-inflammatory M1-like polarization (8). The other immune subsets, including T lymphocytes and neutrophils, also contribute significantly to vascular inflammation and remodeling, thereby accelerating disease development.

Thymic stromal lymphopoietin (TSLP) is an essential cytokine initially recognized for the role in promoting T helper 2 (Th2) responses in asthma and has been validated as a therapeutic target in allergic diseases (9). Structurally related to interleukin-7 (IL-7), TSLP shares the IL-7 receptor α chain (IL-7Rα) as part of its receptor complex (10). Beyond its effects on dendritic cells and T cells, TSLP can directly influence innate immunity by stimulating innate lymphoid cells (ILC) to produce IL-4 and IL-13, thereby amplifying type 2 inflammation (11).

Notably, our previous work revealed a protective role for TSLP in promoting repair after myocardial infarction, indicating pleiotropic functions beyond allergic inflammation (12). Despite its established immunomodulatory roles, the potential involvement of TSLP in AAA pathogenesis remains entirely unexplored. Therefore, this study aims to investigate the specific function and mechanism of TSLP/TSLPR signaling in AAA formation and progression.

## 2 Methods

### 2.1 UK Biobank database analysis

This study utilized individual-level prospective cohort data from the UK Biobank, which includes plasma proteomic data detected by the Olink platform (13). We strictly excluded individuals with diseases known to cause significant elevation of TSLP levels, including atopic dermatitis (ICD-10 L20), asthma (ICD-10 J45), allergic rhinitis (ICD-10 J30), and urticaria (ICD-10 L50) (14). Additionally, based on ICD-10 diagnostic codes and self-reported medical history, we further excluded individuals with various cardiovascular diseases (such as coronary heart disease, heart failure, hypertension, and arrhythmia). This exclusion strategy was designed to minimize, as much as possible, the interference of known non-AAA-related factors on circulating TSLP levels. Second, regarding the quantification of TSLP, we utilized high-throughput proteomics data from the Olink platform. The raw data from the Olink database were normalized using the Olink NPX Signature software and ultimately output as NPX (Normalized Protein Expression) values, which represent the relative expression level of serum TSLP. NPX is a relative quantification unit unique to the Olink platform, scaled on a log_2_ basis. The value reflects the relative abundance of the target protein in the sample rather than its absolute concentration. To mitigate the influence of extreme outliers, we followed Olink database recommendations and included only data with serum TSLP relative expression levels within the 10th to 90th percentile range. We provided a summary of baseline characteristics of the included population in the **Supplementary Materials** (**Supplementary Table 1**). The part of this study was conducted in UK Biobank under the application number 91074.

### 2.2 Experimental animals

Male C57BL/6J mice (10–12 weeks old) were purchased from Beijing Vital River Laboratory Animal Technology (Beijing, China). As per our prior research, Cyagen Bioscience INC (Nanjing, China) generated TSLPR knockout (Tslpr^-/-^) mice with a C57BL/6J genetic background (12). Only male mice were utilized in our research. All animals were randomly assigned to experimental groups. All procedures were approved by the Animal Care and Utilization Committee of Huazhong University of Science and Technology and conducted in accordance with NIH guidelines (12). Mice were anesthetized with 1% sodium pentobarbital (50 mg/kg body weight) via intraperitoneal injection. At the experimental endpoints (e.g., day 14 or day 7 post-operation), mice were humanely euthanized. Euthanasia was performed by cervical dislocation under deep anesthesia to minimize suffering. All efforts were made to minimize animal suffering throughout the study.

### 2.3 AAA induction models

Elastase model: AAA was induced in 10-12-week-old mice using porcine pancreatic elastase (PPE) as previously described (15). We anesthetized mice with pentobarbital sodium (50 mg/kg). Following anesthesia, the infrarenal aorta was isolated and perfused normal saline (sham control) or 10 μl PPE (#E1250, Sigma-Aldrich, USA) for 10 minutes (16).

Calcium phosphate (Ca_3_(PO_4_)_2_) model: AAA was induced in 10-12-week-old mice as previously described (15). We anesthetized mice with pentobarbital sodium (50 mg/kg). Those infrarenal abdominal aorta was isolated and wrapped with gauze soaked in CaCl_2_ for 10 minutes, followed by replacement with PBS for 5 minutes.

After that, we euthanized mice model 7 days (Ca_3_(PO_4_)_2_ model) or 14 days (elastase model) post-operation (16). Then we harvested and photographed the mice abdominal aorta. Maximum external aortic diameter was measured using Image-Pro Plus software, with at least three measurements per mouse averaged before group mean calculation. AAA was defined as ≥50% aortic diameter increase compared to sham controls (16).

### 2.4 Drug treatment strategy in mice

Exogenous TSLP treatment: Recombinant mouse TSLP protein (HY-P70626, MCE, USA) was administered via intraperitoneal injection starting on day 1 after PPE induction at a dose of 20 mg/kg, and then every other day thereafter.

Anti-TSLP antibody treatment: Anti-TSLP antibody (HY-P990150, MCE, USA) was administered via intraperitoneal injection starting on day 1 after PPE induction at a dose of 20 mg/kg, and then every other day thereafter.

### 2.5 Flow cytometry

All cell isolation procedures were performed on ice. Single-cell suspensions were prepared from whole aortas by mincing tissues into fine fragments followed by enzymatic digestion in Aorta Dissociation Enzyme solution (Sigma-Aldrich, USA) at 37 °C for 1 hour as previous described (16). The suspension was then passed through a 70-μm cell strainer for filtration (#322350, BD Biosciences, USA) to remove undigested tissue fragments following the methods of our prior research (16). On ice, cells underwent a 30-minute incubation with fluorochrome-conjugated antibodies for surface marker staining. We used Intracellular Fixation and Permeabilization Buffer Kit from eBioscience (#00552300, USA) to fix and make the cells permeable, strictly following the provided protocol. Once treated, the cells were ready for staining with antibodies directed at intracellular targets.

### 2.6 Histological and immunohistochemical analysis

Aortas were fixed in 4% paraformaldehyde (PFA) for one day, immersed in paraffin and then sectioned (4 μm). We analyzed serial sections from the maximal dilation site were. Elastin integrity was assessed using elastica van Gieson staining (#115974, Sigma–Aldrich, USA) and graded as: 1 (<25% degradation), 2 (25-50%), 3 (50-75%), or 4 (>75%).

Immunostaining was performed using antibodies against TSLP (ab188766, Abcam, UK), CD68 (14-0681-82, Thermo Fisher Scientific, USA) MMP-2 (436000, Invitrogen, USA) and MMP-9(MA5-15886, Invitrogen, USA). We quantified TSLP, CD68, MMP-2, and MMP-9 expression as percentage positive area. All slides were examined using microscope (BX51 OLYMPUS, Japan) by two independent, blinded investigators. 5~6 mice per group were randomly selected for analysis, with ≥3 sections per animal evaluated.

### 2.7 Immunofluorescence

The staining protocol began with permeabilization of sections using 0.3% Triton X-100 (Solarbio, China). And then blocking with 5% FBS (Procell, #164250, China), they were incubated with a mix of primary antibodies (against TSLP, CD45, vimentin, and α-SMA) for 2 hours at normal temperature. Following incubation with fluorophore-conjugated secondary antibody, nuclei were visualized with DAPI.

### 2.8 RT-PCR

To assess gene expression, we first froze aortic tissues in liquid nitrogen and then stored at -80 °C. We performed total RNA isolation with TRIzol reagent (Invitrogen, #15596018, USA). To RNA concentration measurement, we using the NanoDrop spectrophotometer (Thermo Fisher Scientific, USA). After that, cDNA was generated theough the HiScript III RT SuperMix set (Vazyme, #R323, China). Finally, qPCR was carried out in duplicate using Master Mix (Vazyme, #Q711-02, China) on the CFX Connect instrument (Bio-Rad, USA), with expression normalized to Gapdh (2^–ΔΔCT method). The primer sequences used are listed below:

Tslp – Forward: 5′-GGTTCTTCTCAGGAGCCTCTTCATC-3′,

Reverse: 5′-AGGGCAGCCAGGGATAGGATTG-3′;

Gapdh – Forward: 5′-AGAAGGTGGTGAAGCAGGCATC-3′,

Reverse: 5′-CGAAGGTGGAAGAGTGGGAGTTG-3′.

### 2.9 CyTOF staining and acquisition

We prepared single-cell suspensions as previously described (16). We first stained cells with Rh103 intercalator (500 μM; Fluidigm, USA) to discriminate non-viable cells. Subsequently, samples were incubated with metal-tagged antibody panels for mass cytometry. Following surface and intracellular staining, we labeled subsets with Ir191/193 intercalator (Fluidigm, USA) for DNA content detection. Data were acquired on the equipment called Helios2 Mass Cytometer (Fluidigm, USA) with signal normalization by EQ™ Four Element Calibration Beads (Fluidigm, USA).

Normalized FCS files were imported into Cytobank for preliminary analysis. CD45^+^ viable single cells were identified and subpopulations were gated. These gated populations were exported and processed through FlowJo to ensure channel consistency. Using the cytofkit R package, we performed unsupervised clustering via FlowSOM and dimensionality reduction by t-distributed stochastic neighbor embedding (t-SNE), analyzing only markers common across all samples.

### 2.10 Bulk RNA sequencing

Library Preparation and Sequencing: We extracted total RNA and its integrity checked by agarose gel electrophoresis. We determined RNA concentration and quality using the Agilent 2100 Bioanalyzer with the RNA Nano 6000 Assay kit. After enriching mRNA with poly-T magnetic beads and synthesizing cDNA, we constructed libraries. The AMPure XP system was used to select cDNA fragments of 370–420 bp. We assessed library quality with a Qubit 2.0 Fluorometer and confirmed insert size on the Agilent 2100 Bioanalyzer. All libraries were sequenced on the Illumina NovaSeq 6000 for 125/150 bp paired-end reads.

Bioinformatic Analysis: First, raw data were trimmed and filtered with FASTP (v0.19.4) to yield clean reads, with Q20, Q30, and GC content calculated. Next, clean reads were aligned to the reference genome using HISAT2 (v2.0.5). Gene expression quantification was then performed with featureCounts (v1.5.0-p3), and values were normalized using FPKM. Finally, differential gene expression analysis was carried out using DESeq2 (v1.20.0) in R., with adjusted *P*-values (Benjamini–Hochberg method) ≤ 0.05 and |log_2_(foldchange)| ≥ 0.5 set as the significance threshold. Principal component analysis and sample correlation analysis were conducted using ggplot2 (v3.0.3). The Functional enrichment analysis of DEG was performed using the clusterProfiler package (v3.8.1) for Kyoto Encyclopedia of Genes and Genomes (KEGG) pathways and Gene Ontology (GO) terms.

### 2.11 Statistical analysis

Data are presented as mean ± SEM. For small-sample animal and cell experiments, normality was assessed using the Shapiro-Wilk test. For the large-scale UK Biobank dataset, normality was assessed using the Kolmogorov-Smirnov test. Given the non-normal distribution of TSLP levels, the Mann-Whitney U test was used for group comparisons. Homogeneity of variances for multiple group comparisons was evaluated using Levene’s test. For comparisons between two groups, an unpaired two-tailed Student’s t-test was used for parametric data, and the Mann-Whitney U test for non-parametric data. For comparisons among multiple groups, one-way ANOVA followed by Bonferroni’s *post hoc* test was applied. A p-value < 0.05 was considered statistically significant. All statistical analyses were performed using GraphPad Prism (version 10.0).

## 3 Results

### 3.1 TSLP is upregulated in both human and murine AAA

**Figure 1:**
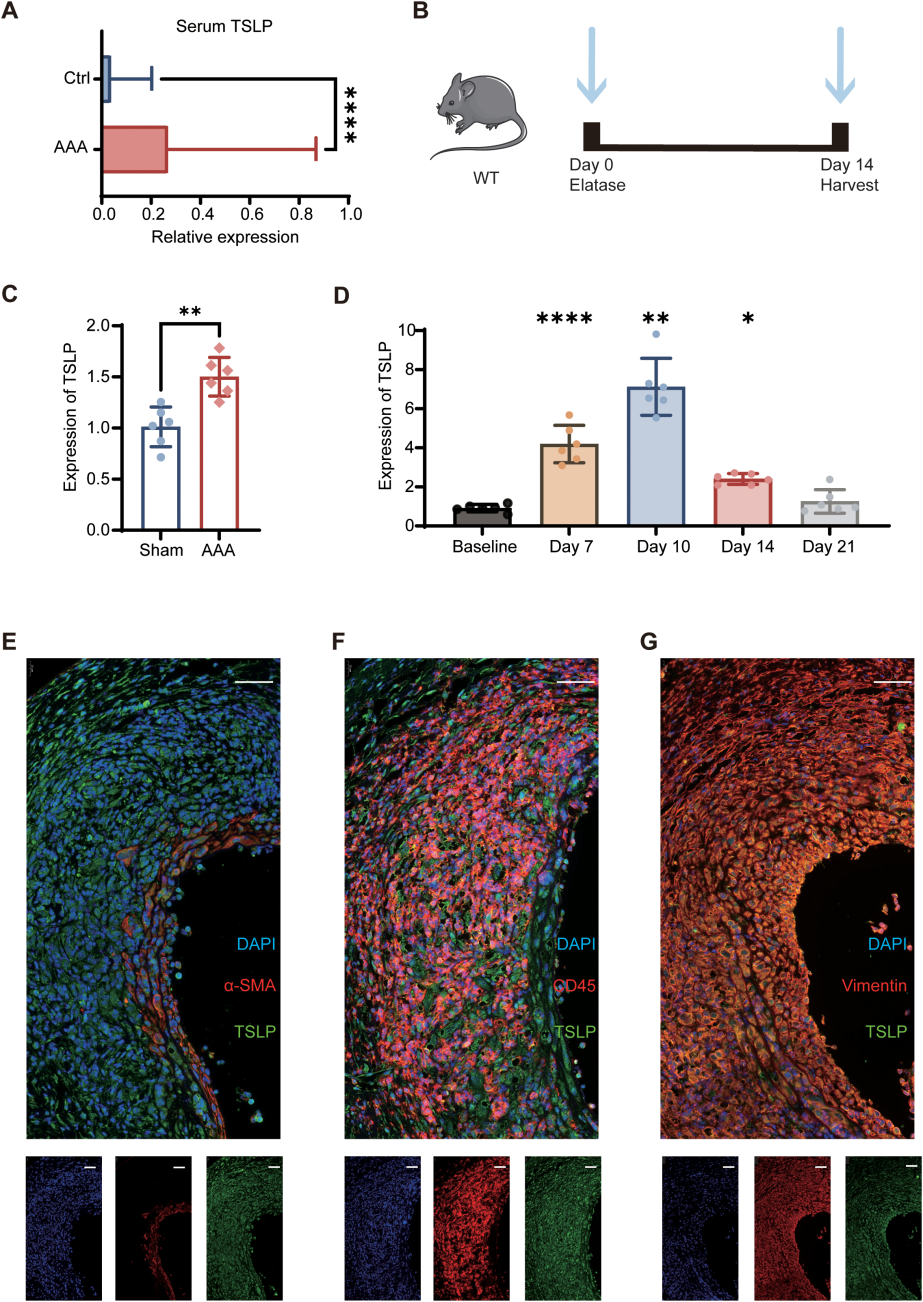
The Expression and origin of TSLP in AAA. **(A)** Serum TSLP concentrations patients with AAA (n=632) compared with healthy controls (n=24,036) in UKB. **(B)** The schematic diagram is presented below for reference. 10-12-week- aged male mice received a PPE induction at day 0, with aortic sampling performed at day 14. **(C)** The comparison of TSLP mRNA expression in the aortas from the sham and AAA groups at day 14 (n=6 mice per group). **(D)** Temporal expression profile of TSLP mRNA in the murine aorta at indicated time points after AAA induction, compared to the baseline level (n=6 mice per group). **(E-G)** Representative immunofluorescence images of murine AAA sections stained for TSLP (red), cell-specific markers (green), and DAPI (blue). **(E)** Co-staining with CD45 (leukocyte marker). **(F)** Co-staining with vimentin (fibroblast marker). **(G)** Co-staining with α-SMA (smooth muscle cell marker) (n=4 mice per group). Data are presented as mean ± SEM. Scale bar: 50 μm. *p < 0.05, **p < 0.01, ***p < 0.001, ****p < 0.0001 (the Mann–Whitney U test was used in A, unpaired two-tailed Student’s t-test was used in C, and one-way ANOVA was used in D).

We first investigated the clinical relevance of TSLP with AAA in human. Interrogation of the UK Biobank (UKB) dataset demonstrated a significant elevation in serum TSLP levels in AAA patients (**Figure 1A**). We next sought to delineate the spatial and temporal expression of TSLP within the aortic wall using the well-established porcine pancreatic elastase (PPE)-induced murine AAA model (**Figure 1B**). RT-PCR analysis revealed a dynamic time-course of TSLP mRNA expression, which was significantly induced post-induction, peaked at day 10, and remained substantially elevated at day 14 (**Figures 1C, D**). To pinpoint the cellular origin of TSLP in the aneurysmal milieu, we performed immunofluorescence co-staining. The results identified fibroblasts as the predominant source of TSLP protein. Less contributions were detected from infiltrating immune cells and vascular smooth muscle cells (**Figures 1E–G**).

### 3.2 TSLPR deficiency confers protection against experimental AAA formation

**Figure 2:**
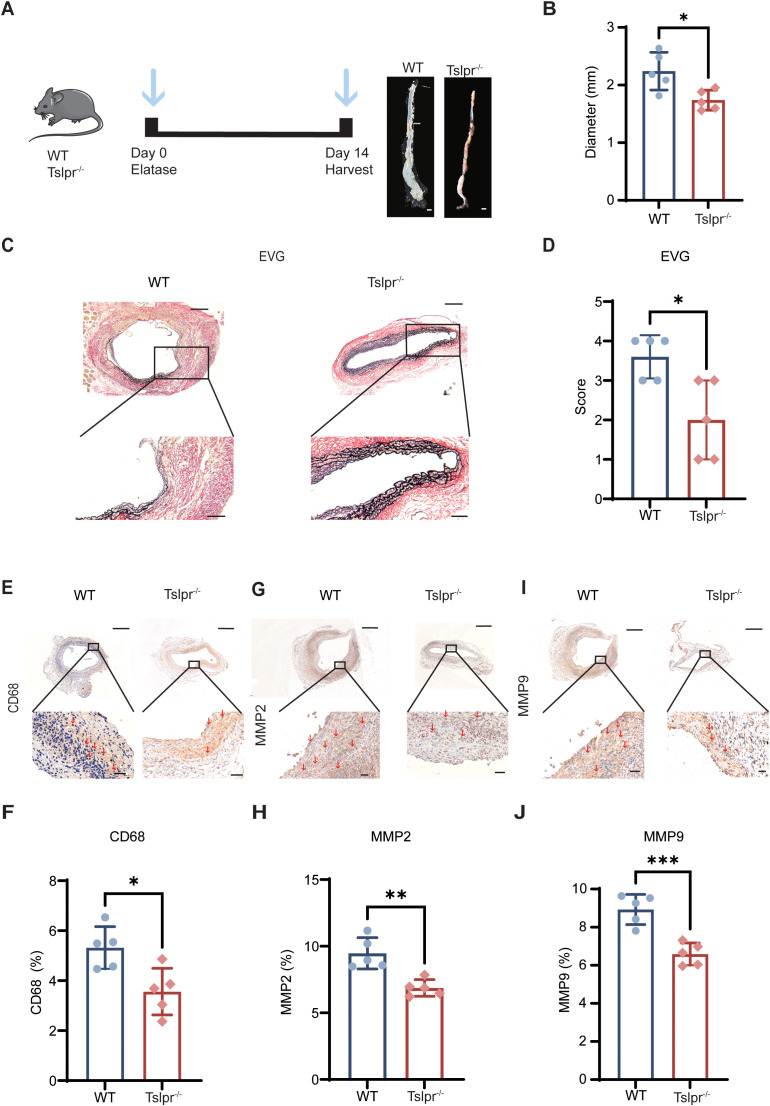
Genetic ablation of TSLP receptor attenuates experiment al AAA severity. **(A)** Schematic of the experimental design utilizing wildtype (WT) and Tslpr^−^/^−^ mice in the PPE-induced AAA model. **(B)** Quantification of the maximum abdominal aortic diameter in WT and Tslpr^−^/^−^ mice at day 14 post-PPE induction (n=5 mice per group). **(C)** Representative images of elastic Elastica Van Gieson (EVG) staining of aortic tissue. **(D)** Elastin degradation scores evaluated by EVG staining (n=5 mice per group). **(E–J)** Representative immunohistochemical staining and quantitative analysis of CD68 **(E, F)**, MMP2 **(G, H)** and MMP9 **(I, J)** expression in the aortic wall. Positive areas are presented as a percentage of the total area (n=5 mice per group). Scale bars: **(A)** 1mm; **(C)** 100 μm (top), 50 μm (bottom); **(E, G, I)** 500 μm (top), 40 μm (bottom). All quantitative data are presented as mean ± SEM. *p < 0.05, **p < 0.01, ***p < 0.001 (unpaired two-tailed Student’s t-test). Red arrows indicate representative positive staining areas.

To directly investigate the functional impact of TSLP signaling in AAA, we subjected Tslpr^−^/^−^ mice and WT mice to the PPE-induced AAA model (**Figure 2A**). The absence of TSLP signaling conferred a marked protection against aortic dilation, as evidenced by a significantly reduced maximum aortic diameter in Tslpr^−^/^−^ mice compared to WT controls at day 14 (**Figure 2B**).

Consistent with this macroscopic protective effect, histological analysis revealed substantial mitigation of key pathological features in Tslpr^−^/^−^ aortas. Aortic tissues exhibited lower elastin degradation scores, as quantified by Elastica Van Gieson (EVG) staining (**Figures 2C, D**). Furthermore, infiltration of CD68^+^ macrophages in the aorta was notably decreased in Tslpr^−^/^−^ mice (**Figures 2E, F**). Given the pivotal role of matrix metalloproteinases (MMPs) in elastin breakdown (17), we assessed the expression of MMP2 and MMP9. Immunohistochemical analysis demonstrated a significant reduction in the positive areas for both MMP2 (**Figures 2G, H**) and MMP9 (**Figures 2I, J**) within the aneurysmal lesions of Tslpr^−^/^−^ mice.

To substantiate these findings in an independent model, we employed a Ca_3_(PO_4_)_2_-induced AAA model. The results from this complementary approach consistently recapitulated the attenuated AAA phenotype in Tslpr^−^/^−^ mice (**Supplementary Figure 1**). Collectively, these data from two distinct experimental models demonstrate that genetic disruption of the TSLP/TSLPR pathway significantly mitigates the development and severity of AAA.

### 3.3 Therapeutic inhibition of TSLP protects against PPE-induced AAA

**Figure 3:**
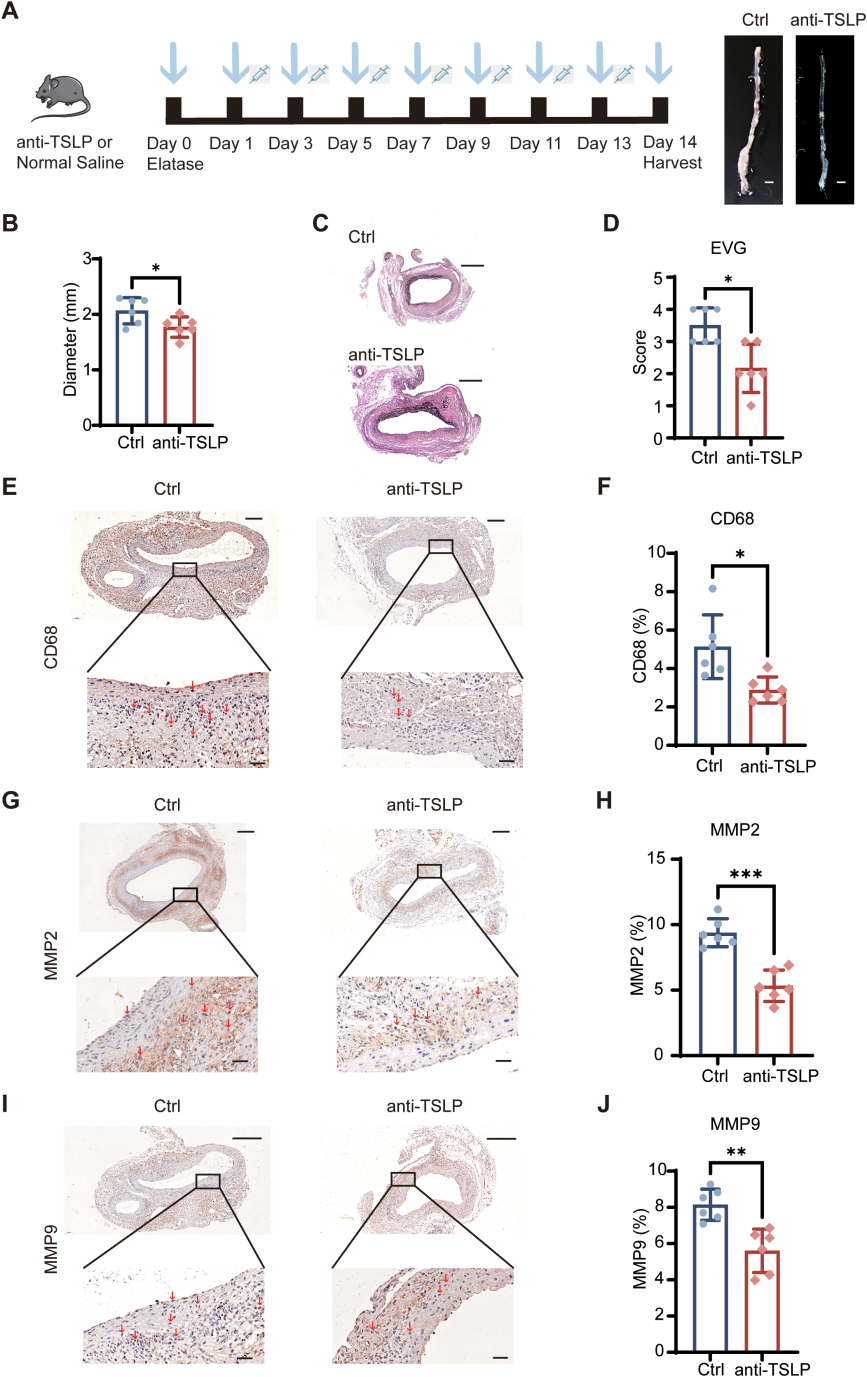
Therapeutic neutralization of TSLP protects against AAA development. **(A)** Schematic diagram of the experimental protocol for anti-mouse TSLP antibody (anti-TSLP) administration in the PPE-induced AAA model. **(B)** Comparison of the maximum abdominal aortic diameter in normal saline-treated and TSLP-treated mice at day 14 (n=6 mice per group). **(C, D)** Representative histological staining and corresponding quantification for Elastica Van Gieson (EVG) (n=6 mice per group). **(E–J)** Representative images and quantitative analysis of immunohistochemical staining for CD68 **(E, F)**, MMP2 **(G, H)** and MMP9 **(I, J)** expression in the aortic wall. Positive areas are presented as a percentage of the total area (n=6 mice per group). Scale bars: **(A)** 1mm; **(C)** 500 μm; **(E, G, I)** 200 μm (top), 50 μm (bottom). All quantitative data are presented as mean ± SEM. *p < 0.05, **p < 0.01, ***p < 0.001 (unpaired two-tailed Student’s t-test). Ctrl, control. Red arrows indicate representative positive staining areas.

Given the observed protection in Tslpr^−^/^−^ mice, we investigated the therapeutic potential of TSLP inhibition using a monoclonal antibody in the PPE-induced AAA model. Inspired by the clinical success of Tezepelumab, a human anti-TSLP antibody approved for severe asthma (18), we administered a specific anti-mouse TSLP antibody (anti-TSLP) to mice following AAA induction (**Figure 3A**).

Anti-TSLP treatment substantially alleviated the progression of AAA, as demonstrated by a reduction in aortic dilation compared to the normal saline-treated control group (**Figure 3B**). Histological evaluation revealed that anti-TSLP preserved the structural integrity of the aortic wall, which manifested as protection against elastin fragmentation (**Figures 3C, D**). Consistent with these findings, the antibody-treated group showed a marked drop in the expression of MMP2 and MMP9, as well as a decrease in CD68^+^ macrophage infiltration (**Figures 3E–J**). These results indicate that anti-TSLP mitigates inflammatory infiltration and extracellular matrix degradation in the aortic wall. Collectively, the data from both genetic and pharmacological interventions robustly demonstrate that targeting the TSLP pathway effectively alleviates experimental AAA.

### 3.4 Exogenous TSLP administration aggravates PPE-induced AAA

**Figure 4:**
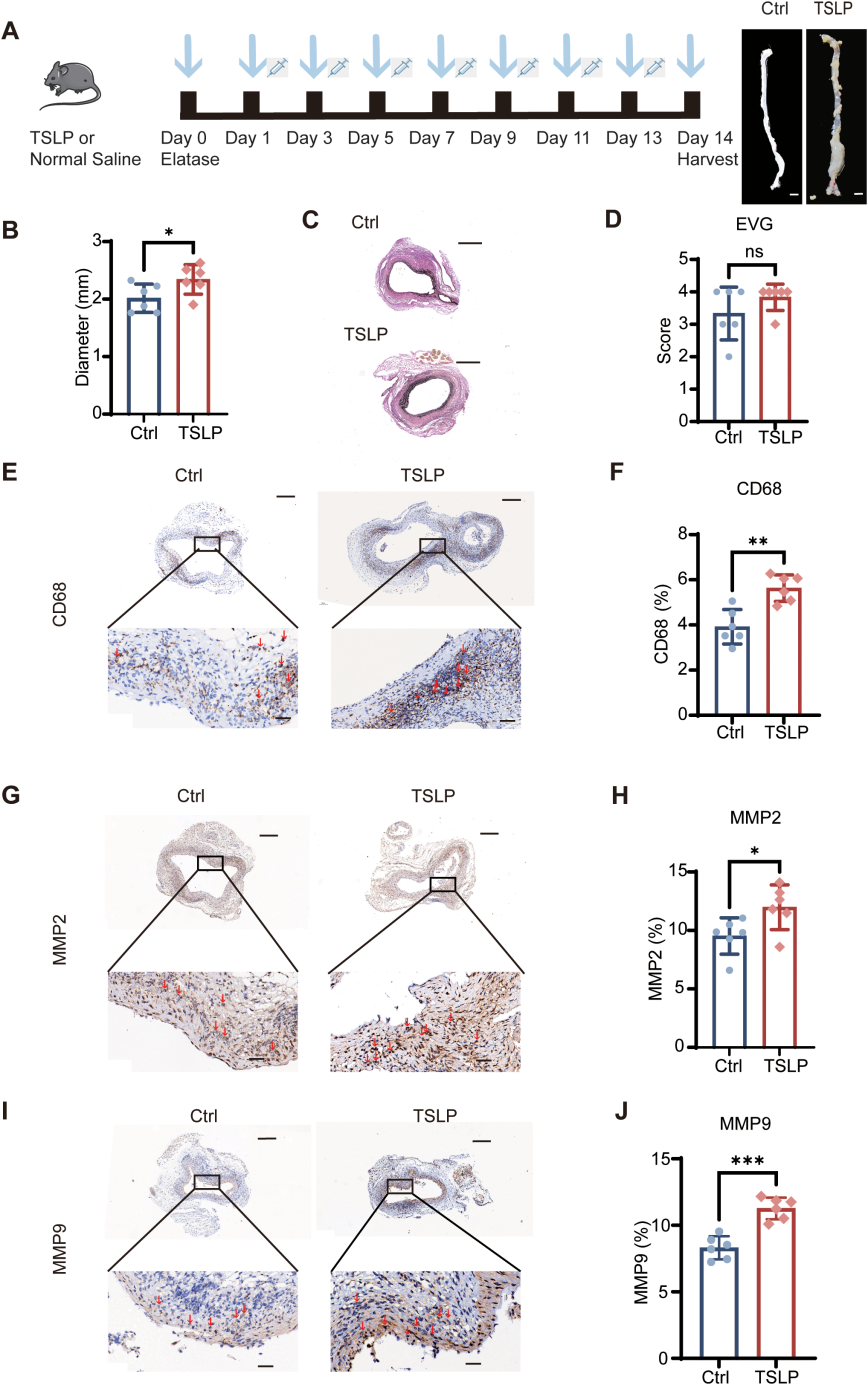
Exogenous TSLP administration aggravates AAA in mice. **(A)** Schematic diagram of the experimental protocol for recombinant TSLP administration in the PPE-induced AAA model. **(B)** Comparison of the maximum abdominal aortic diameter in normal saline-treated and TSLP-treated mice at day 14 (n=6 mice per group). **(C, D)** Representative histological staining and corresponding quantification for EVG (n=6 mice per group). **(E–J)** Representative images and quantitative analysis of immunohistochemical staining for CD68 **(E, F)**, MMP2 **(G, H)** and MMP9 **(I, J)** expression in the aortic wall. Positive areas are presented as a percentage of the total area (n=6 mice per group). Scale bars: **(A)** 1mm; **(C)** 500 μm; **(E, G, I)** 200 μm (top), 40 μm (bottom). All quantitative data presented as mean ± SEM. *p < 0.05, **p < 0.01, ***p < 0.001, ns, no significant difference (unpaired two-tailed Student’s t-test). Ctrl, control. Red arrows indicate representative positive staining areas.

To provide complementary evidence for the pathogenicity of TSLP, we assessed the effect of its exogenous administration. Recombinant TSLP was administered to mice following PPE-induced AAA induction (**Figure 4A**), which significantly exacerbated aortic dilation compared to normal saline-treated controls (**Figure 4B**).

Notably, while the degrees of elastin fragmentation were comparable between groups (**Figures 4C, D**), TSLP treatment markedly intensified the inflammatory response. This was evidenced by a great increase in the expression of MMP2 and MMP9, alongside enhanced infiltration of CD68^+^ macrophages within the aortic wall (**Figures 4E–J**). Collectively, these data demonstrate that elevating TSLP levels worsens experimental AAA, primarily by amplifying inflammation and protease activity, rather than by directly altering the structural composition of the matrix at the endpoint.

### 3.5 TSLP regulates macrophage M1/M2 polarization

**Figure 5:**
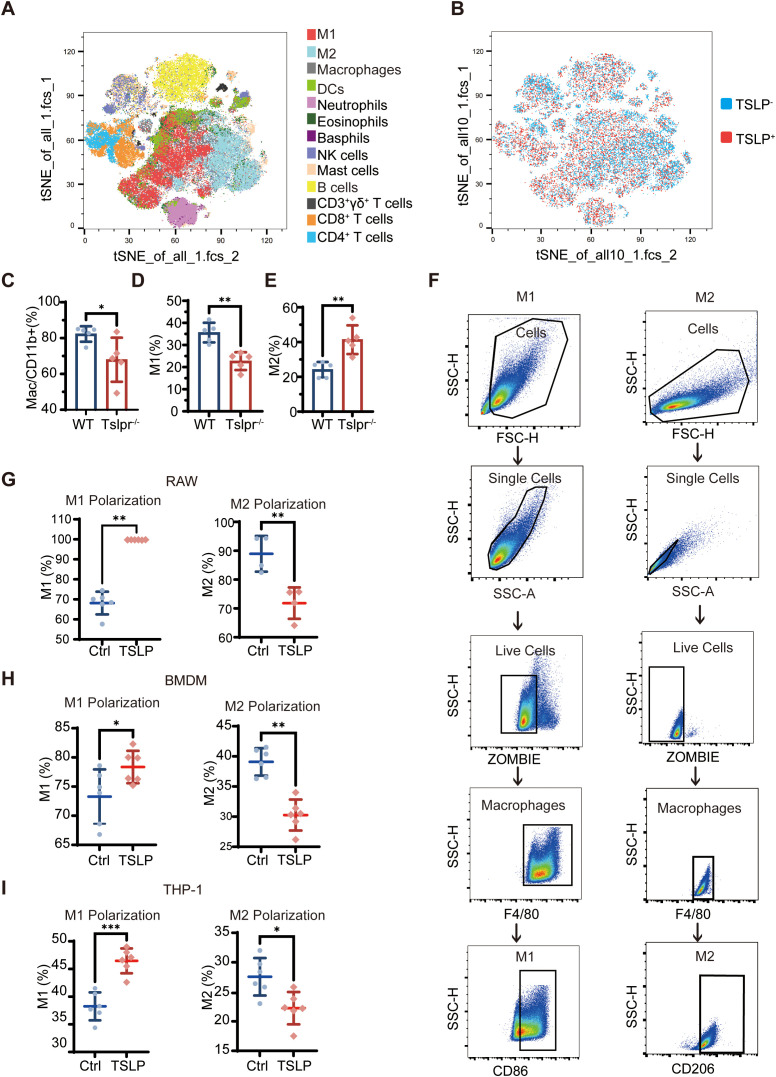
TSLP signaling regulates the immune microenvironment and macrophage polarization in AAA. **(A, B)** viSNE plots visualizing the high-dimensional CyTOF data, depicting the overall immune cell composition and the specific distribution of TSLP. **(C)** Quantitative analysis of the total macrophage frequency among CD11b^+^ immune cells in the aortic tissues. **(D, E)** The ratio of M1 to M2 macrophages in the aortic tissues of WT and Tslpr^−^/^−^ mice. **(F)** Gating strategy for M1 (CD86^+^) and M2 (CD206^+^) macrophages in RAW264.7 cells and BMDMs by flow cytometry. **(G, H)** Flow cytometry analysis and quantification of the percentages of M1 (CD86^+^) and M2 (CD206^+^) macrophages in RAW264.7 cells **(G)** and BMDMs **(H)** following polarization and TSLP treatment. **(I)** Proportions of M1 and M2 macrophages in PMA-differentiated THP-1 cells treated with or without TSLP. Cell stimulation conditions: M1 polarization control (Ctrl), 100ng/ml LPS + 20ng/ml IFN-γ for 24h; M1 polarization with TSLP (TSLP), 100ng/ml LPS + 20ng/ml IFN-γ+20ng/ml TSLP for 24h; M2 polarization control (Ctrl), 20ng/ml IL-4 for 24h; M2 polarization with TSLP (TSLP), 20ng/ml IL-4 + 20ng/ml TSLP for 24h (n=4–6 per group). Data are presented as mean ± SEM. *p < 0.05, **p < 0.01, ***p < 0.001 (unpaired two-tailed Student’s t-test). M1, M1 macrophages; M2, M2 macrophages; DCs, dendritic cells; NK cells, natural killer cells.

To systemically characterize the immune landscape modulated by TSLP signaling, we performed CyTOF mass cytometry on aortic tissues from PPE-induced WT and Tslpr^−^/^−^ mice, utilizing a panel of 45 leukocyte markers (**Figures 5A, B**; **Supplementary Table 2**). viSNE analysis of 457,869 single cells revealed a significant alteration in the macrophage compartment upon Tslpr deletion. Specifically, Tslpr^−^/^−^ mice exhibited a less overall proportion of macrophages. More importantly, this shift was accompanied by a decreased of pro-inflammatory M1 macrophages (CD86^+^CD206^−^) and a concurrent increase in reparative M2 macrophages (CD86^−^CD206^+^) (**Figures 5C–E**).

We next sought to determine whether TSLP directly regulates macrophage polarization. Using RAW264.7 cells and mouse bone marrow-derived macrophages (BMDMs)—the identity of which was confirmed by high expression of F4/80 via flow cytometry (**Figure 5F**)—we established that TSLP (20 ng/mL) exerted a direct polarizing effect. In cells pre-stimulated with interferon γ(IFN-γ) + lipopolysaccharide (LPS) or IL-4, TSLP treatment significantly elevated the proportion of CD86^+^ M1 macrophages and declined the proportion of CD206^+^ M2 macrophages (**Figures 5G, H**). This TSLP-driven shift toward a pro-inflammatory state was consistently reproduced in PMA-differentiated THP-1 cells (**Figure 5I**; **Supplementary Figure 2A**). Critically, TSLP had no effect on the polarization of unprimed RAW264.7 or THP-1 cells (**Supplementary Figures 2B, C**), indicating that its function was context-dependent and required a pre-existing inflammatory signal.

Collectively, these findings from both *in vivo* and *in vitro* models demonstrate that TSLP not only promotes M1 polarization but also actively suppresses M2 polarization, thereby skewing macrophages toward a pro-inflammatory phenotype.

### 3.6 Transcriptomic profiling reveals TSLP-mediated activation of pro-inflammatory pathways in macrophages

**Figure 6:**
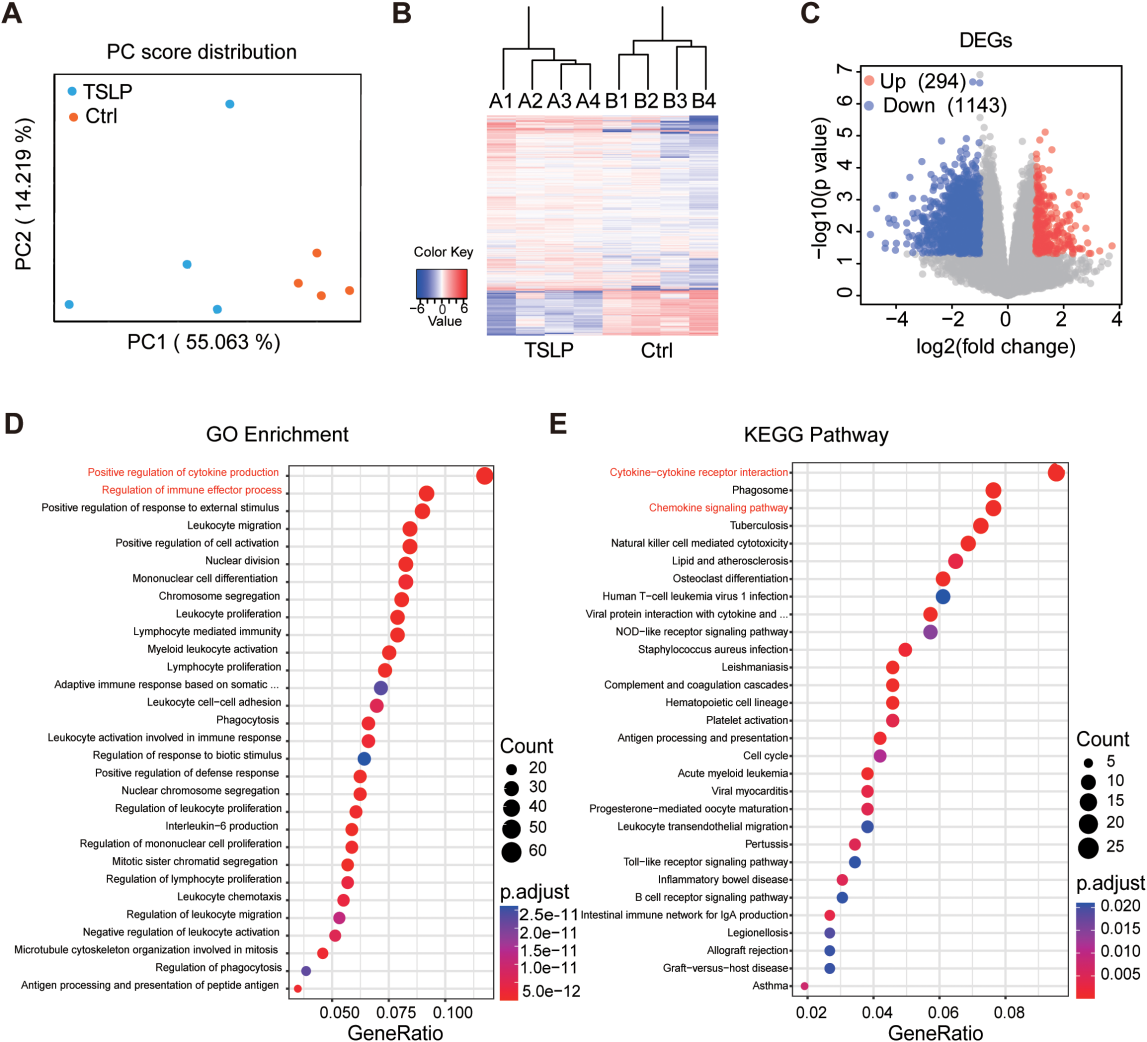
Transcriptomic profiling reveals TSLP-mediated activation of pro-inflammatory pathways in macrophages. RAW 264.7 was stimulated with 100ng/ml LPS + 20ng/ml IFN-γ+20ng/ml TSLP (TSLP group) or 100ng/ml LPS + 20ng/ml IFN-γ for 24h (Control group) and then subjected to RNA-sequencing. **(A)** Principal component analysis (PCA). **(B)** Clustering diagram of differentially expressed gene (DEG) pattern. **(C)** Volcano plot displays DEGs. **(D, E)** Gene Ontology (GO) enrichment and Kyoto Encyclopedia of Genes and Genomes (KEGG) pathway analyses of downregulated genes. Data are from n=4 samples per group.

To elucidate the transcriptomic mechanisms underlying TSLP-driven macrophage polarization, we performed bulk RNA sequencing on LPS+IFN-γ-primed RAW264.7 cells treated with or without exogenous TSLP. TSLP treatment induced significant alterations in the cellular transcriptomic profile (**Figures 6A, B**), resulting in the upregulation of 294 genes and the downregulation of 1,143 genes (**Figure 6C**). Gene Ontology (GO) and KEGG pathway analysis demonstrated that the DEGs were considerable enriched in pathways critical to AAA pathogenesis, including positive regulation of cytokine production, regulation of immune effector process, cytokine−cytokine receptor interaction and chemokine signaling pathway (**Figures 6D, E**). Collectively, these transcriptomic data demonstrate that TSLP directly instigates a broad pro-inflammatory program in macrophages *in vitro*, characterized by enhanced cytokine signaling pathway. This cell-intrinsic mechanism provides a plausible molecular basis for the accelerated AAA progression observed upon TSLP administration *in vivo*.

## 4 Discussion

This study identifies TSLP as a previously unrecognized promoter of AAA pathogenesis. By integrating evidence from human biobank data, multiple murine models, and *in vitro* assays, we demonstrate that TSLP exacerbates AAA progression by directly promoting pro-inflammatory M1 macrophage polarization and inhibiting reparative M2 polarization. Our initial clinical association analysis revealed elevated serum TSLP levels in AAA patients, positioning TSLP as a relevant biomarker in human disease. Subsequent mechanistic investigations in mice established a direct causal role for the TSLP/TSLPR pathway. The attenuated AAA phenotype observed in Tslpr^−^/^−^ mice unequivocally demonstrated that TSLP signaling is necessary for full disease manifestation. This conclusion is further strengthened by our pharmacological studies, which showed that neutralizing TSLP therapeutically protected against AAA, while exogenous TSLP administration exacerbated disease severity.

TSLP is primarily produced by stromal and epithelial cells in tissues such as the skin and lung (19), with a notable and specific expression within the epithelial cells of Hassall’s bodies in the thymus (20). Under pathological conditions, its production extends to other subsets such as dendritic cells, fibroblasts, mast cells and hepatocytes (21, 22). Our study identified fibroblasts as the primary cellular source of TSLP within AAA. This finding aligns with the established role of fibroblasts as active immune modulators in various inflammatory settings. For instance, in pulmonary inflammation induced by mechanical injury, fibroblasts secrete lots of cytokines, such as IL-1, IL-4, IL-6, IL-8, IL-13 and TSLP (23). These cytokines critically regulate the recruitment, trafficking, and polarization of diverse immune subsets, such as macrophages, neutrophils, eosinophils, natural killer (NK) cells, and multiple T-lymphocyte population, comprising regulatory T cells (Tregs), Th1, Th2, and Th17. Similarly, in intestinal mucosa, fibroblasts sustain immune homeostasis by constitutively secreting TSLP, which regulates the innate lymphoid cell type 2 (ILC2)–tuft cell circuit (24). Furthermore, Cui et al. demonstrated that fibroblasts in the tumor microenvironment secrete substantial TSLP to impede CD8^+^ T-cell activity (25). Collectively, these observations position TSLP as a novel and significant component of the fibroblast secretome, suggesting that fibroblast-derived TSLP may serve as a critical linchpin linking vascular wall stress to the ensuing immune response in AAA. Concurrently, our data also indicate a minor but notable contribution from CD45^+^ hematopoietic cells. Within the AAA milieu, macrophages constitute the most abundant population of inflammatory leukocytes. Studies have shown that alveolar macrophages can produce TSLP in asthma and pulmonary fibrosis; niche-specific macrophages in other tissues might contribute to local TSLP pools (26, 27). Therefore, it is plausible that a fraction of TSLP in AAA originates from infiltrating macrophages or other CD45^+^ cells, potentially creating an autocrine or paracrine loop that further stabilizes the pro-inflammatory polarization state. The exact identity and functional significance of these TSLP-producing immune cells within the aortic lesion present an intriguing avenue for future investigation.

A central finding of our study is the identification of macrophage polarization as the central cellular mechanism through which TSLP exerts its effect in AAA. In this study, we used CD86 and CD206 as markers to identify M1 and M2 macrophages. This definition is widely accepted in functional studies and allows high-throughput assessment of TSLP effects on macrophage polarization (28). Our high-dimensional CyTOF analysis of the aortic microenvironment revealed that genetic disruption of TSLP signaling reduces the M1/M2 macrophage ratio. The *in vivo* observation was corroborated by direct *in vitro* evidence across multiple macrophage models, where TSLP consistently promoted M1 and suppressed M2 polarization. The transcriptomic profile of TSLP-treated macrophages—marked by upregulation of pathways related to cytokine production, cytokine-cytokine receptor interactions, and myeloid leukocyte activation—provides a molecular foundation for the pro-inflammatory effects of TSLP in AAA. The findings of Qin et al. in allergic conjunctivitis (29) align with and support our results. Conversely, Zhou et al. proposed that TSLP pretreatment promoted M2 polarization (30). Critical differences in the duration and concentration of TSLP exposure across experimental settings likely explain this discrepancy. Intriguingly, a layer of post-translational regulation has been identified. It has been demonstrated that KAT3B desuccinylates TSLP, thereby stabilizing it and subsequently promoting M2 while inhibiting M1 polarization (31). This pivotal finding strongly suggests that the functional output of TSLP is not absolute but can be modulated by its biochemical modification state, such as succinylation, which likely varies across different disease microenvironments. However, we acknowledge that the binary M1/M2 classification represents a simplification of macrophage activation states. It is now widely recognized that macrophage activation exists along a continuum, with M1 and M2 representing extremes of this spectrum rather than discrete subsets (32–34). Future studies employing higher-resolution approaches such as single-cell transcriptomics will provide a more comprehensive view of TSLP-mediated macrophage reprogramming *in situ*. While this study focused on macrophages, our CyTOF analysis revealed that TSLP signaling deficiency also alters the proportions of Tregs, NK cells, and cDCs in the aorta (**Supplementary Figure 3**). Whether these subsets are directly regulated by TSLP and what roles they play in TSLP-driven AAA progression are important scientific questions that merit future investigation. Our transcriptomic analysis revealed TSLP-induced inflammatory gene expression profiles in macrophages, the proximal signaling events downstream of TSLPR remain to be elucidated. Whether STAT5, NF-κB, or MAPK pathways are involved in TSLP-mediated pro-inflammatory polarization in macrophages is a key scientific question warranting further investigation.

Collectively, these observations underscore that the specific mechanisms by which TSLP regulates macrophage polarization are complex and context-dependent, warranting further in-depth investigation.

The contrasting roles of TSLP in different disease contexts—protective in myocardial infarction yet detrimental in AAA—highlight the complex functions of this cytokine (12). In AAA, our data establish that TSLP primarily acts through innate immune reprogramming by driving macrophages toward pro-inflammatory phenotype. The clinical relevance of our findings is supported by the development of Tezepelumab, a human anti-TSLP monoclonal antibody approved for severe asthma (18). Our preclinical data suggest that targeting TSLP could represent a viable strategy for mitigating the chronic inflammation that fuels AAA progression.

## 5 Study limitations

We acknowledge several limitations. First, in the present analysis of the UK Biobank data, we did not retrieve information on clinical covariates such as smoking status, BMI, hypertension, dyslipidemia, and diabetes. Therefore, multivariable regression adjustment could not be performed, and the potential confounding effects of these factors on the association between TSLP and AAA cannot be entirely excluded. Meanwhile, the precise upstream regulators of TSLP expression in aortic fibroblasts remain to be fully elucidated. Besides, the identification of TSLP-producing cells in this study was primarily based on the combination of vimentin, α-SMA, and CD45, which may not completely exclude other mesenchymal subsets (e.g., pericytes). Future studies employing more specific fibroblast markers such as PDGFRα or FSP1 are warranted to precisely identify the cellular source of TSLP in AAA tissues (35). Next, our study primarily relied on murine models, and future investigations should include analysis of human AAA tissue samples to validate TSLP expression patterns and cellular sources in human disease. Finally, the relative contribution of macrophage polarization versus other potential immune mechanisms to the overall protective effect of TSLP blockade warrants further investigation.

## 6 Conclusions

In summary, we demonstrate that TSLP promotes AAA progression by driving macrophage polarization toward the M1 phenotype and away from the M2 phenotype. Our findings nominate the TSLP/TSLPR axis as a compelling therapeutic target worthy of further investigation in the management of AAA.

## Data Availability

The datasets presented in this study can be found in online repositories. The names of the repository/repositories and accession number(s) can be found below: PRJNA1440141 (Bioproject, NCBI).
