# Supplementary material for: Thymic stromal lymphopoietin promotes abdominal aortic aneurysm formation by regulating macrophage polarization
Source: Front Immunol. 2026 Apr 7;17:1767913. doi: 10.3389/fimmu.2026.1767913 (PMC13095562; doi:10.3389/fimmu.2026.1767913)
Supplement: Supplementary file 1 [file DataSheet1.pdf]

# *Supplementary Material*

## Supplementary Figures and Tables

**Supplementary Figure 1** Validation in the $Ca_3(PO_4)_2$-induced AAA model.

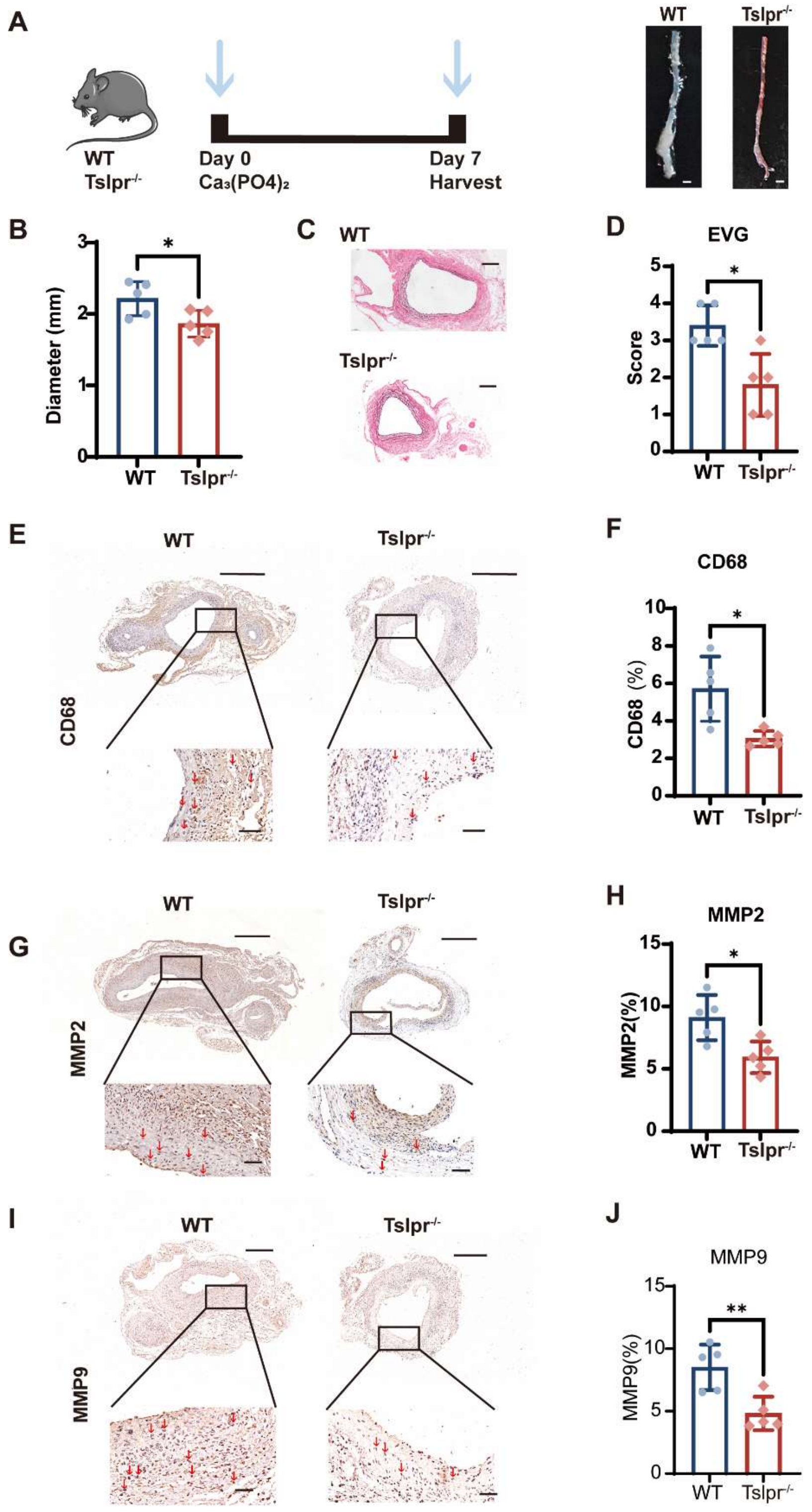


(A) Schematic of the experimental design utilizing wildtype (WT) and Tslpr−/− mice in the $Ca_3(PO_4)_2$-induced AAA model.

(B) Quantification of the maximum abdominal aortic diameter in WT and $Tslpr^{-/-}$ mice at day 7 post-$Ca_3(PO_4)_2$ model induction (n=5 mice per group).
(C) Representative images of elastic Elastica Van Gieson (EVG) staining of aortic tissue.
(D) Elastin degradation scores evaluated by EVG staining (n=5 mice per group).
(E-J) Representative immunohistochemical staining and quantitative analysis of CD68 (E-F), MMP2 (G-H) and MMP9 (I-J) expression in the aortic wall. Positive areas are presented as a percentage of the total area (n=5 mice per group).
Scale bars: (A) 1 mm; (C) 500 μm; (E, G, I) 500 μm (top), 40 μm (bottom). All quantitative data are presented as mean ± SEM. *$p< 0.05$, **$p< 0.01$(unpaired two-tailed Student's t-test).

**Supplementary Figure 2** TSLP has no effect on the polarization of unprimed RAW264.7 or THP-1 cells.

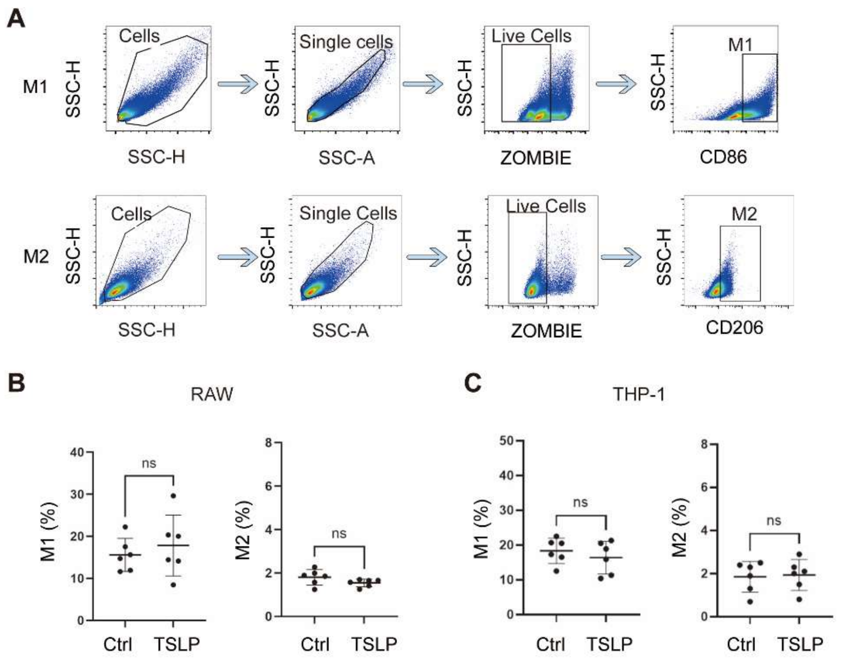


(A) Gating strategy for M1 ($CD86^+$) and M2 ($CD206^+$) macrophages in THP-1 by flow cytometry.
(B, C) Comparative flow cytometry analysis of M1/M2 macrophage polarization ratios in RAW264.7 and THP-1 cells under basal conditions (Ctrl) and upon TSLP treatment (TSLP). Data are presented as mean ± SEM. The data in (B, C) were analyzed by unpaired two-tailed Student's t-test (n=6 per group).

**Supplementary Figure 3** TSLP signaling regulates the immune microenvironment

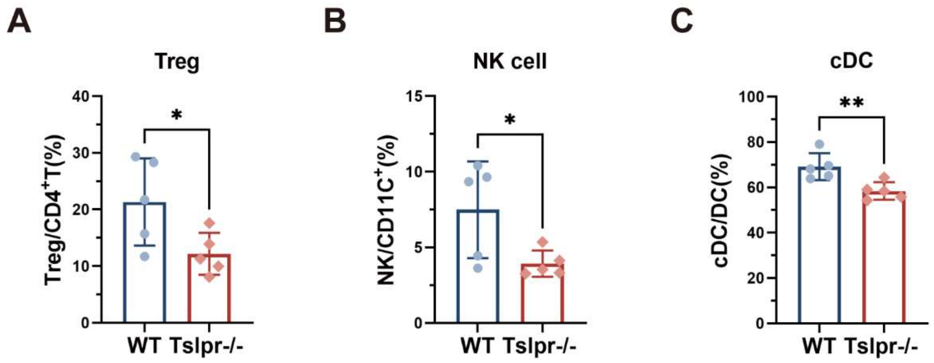


(A) Proportion of regulatory T cells (Tregs) in aortic tissues of WT and Tslpr$^{-/-}$ mice.
(B) Proportion of natural killer (NK) cells in aortic tissues of WT and Tslpr$^{-/-}$ mice.
(C) Proportion of conventional dendritic cells (cDCs) in aortic tissues of WT and Tslpr$^{-/-}$ mice. Data are presented as mean ± SEM. *p< 0.05, **p< 0.01(unpaired two-tailed Student's t-test).

**Supplementary Table 1** Baseline characteristics of abdominal aortic aneurysm patients and non-AAA controls.

| | AAA | Ctrl | p |
|---|---|---|---|
| Age (years) | 64 (60, 67) | 57 (49, 63) | p<0.0001 |
| Male (%) | 78.5 | 43.6 | p<0.0001 |

Age is presented as median (Q1, Q3), and male sex is presented as percentage. The Mann–Whitney U test was used.

**Supplementary Table 2** Summary of the 45-marker antibody panel used for CyTOF analysis of aortic tissues

| Subsets | biomarker |
|---|---|
| Leukocyte | $CD45^{+}$ |
| T cell | $CD3^{+}NK1.1^{-}$ |
| $CD4^{+}$ T cell | $CD3^{+}CD4^{+}CD8\alpha^{-}$ |
| Th1 | $CD3^{+}CD4^{+}CXCR3^{+}CCR4^{-}CCR6^{-}$ |
| Th2 | $CD3^{+}CD4^{+}CXCR3^{-}CCR4^{+}CCR6^{-}$ |
| Th17 | $CD3^{+}CD4^{+}CXCR3^{-}CCR4^{+}CCR6^{+}CD161^{-}$ |
| $T_{fh}$ | $CD3^{+}CD4^{+}CXCR5^{+}$ |
| Treg | $CD3^{+}CD4^{+}CD25^{+}FOXP3^{+}$ |
| $CD4^{+}$ T Naïve | $CD3^{+}CD4^{+}CD44^{low}CD62L^{high}CCR7^{+}$ |

**Supplementary Table 2 Continued**

| Subsets | biomarker |
|---|---|
| $CD4^+$ $T_{CM}$ | $CD3^+CD4^+CD44^{high}CD62L^{high}CCR7^+$ |
| $CD4^+$ $T_{EM}$ | $CD3+CD4^+CD44^{high}CD62L^{low}CCR7^-$ |
| $CD8^+$ T cell | $CD3^+CD4^-CD8\alpha^+$ |
| $CD8^+$ T Naïve | $CD3^+CD8\alpha^+CD44^-CD62L^+CCR7^+$ |
| $CD8^+$ $T_{CM}$ | $CD3^+CD8\alpha^+CD44^+CD62L^+CCR7^+$ |
| $CD8^+$ $T_{EM}$ | $CD3^+CD8\alpha^+CD44^+CD62L^-CCR7^-$ |
| NKT cell | $CD3^+NK1.1^+CD49b^+CD335^+CD11b^-CD19^-$ |
| γδ T cell | $CD3^+TCR\beta^-TCR\gamma\delta^+$ |
| Mast cell | $Fc\varepsilon RI\alpha^+CD117^+$ |
| DC | $CD11c^{high}Ly\text{-}6G^-F4/80^-CD49b^-TCR\beta^-CD3^-CD19^-B220^-$ |
| cDC | $CD11c^{high}MHC\text{-}II^{high}Ly\text{-}6G^-F4/80^-$ |
| pDC | $CD11c^{high}MHC\text{-}II^{high}B220^+Ly\text{-}6C^+CD11b^-CD161^-$ |
| B cell | $B220^+CD19^+Ly\text{-}6G^-$ |
| Plasma cell | $CD138^+IgM^{low}$ |
| Plasmablast | $CD138^-IgM^{high}$ |
| NK cell | $NK1.1^+CD335^+CD49b^+CD3^-TCR\beta^-CD19^-Ly\text{-}6G^-$ |
| Macrophage | $CD11b^+F4/80^+Ly\text{-}6G^-CD335^-CD3^-CD19^-B220^-TCR\beta^-$ |
| M1 | $CD11b^+F4/80^+MHC\text{-}II^{high}CD206^-CD86^+$ |
| M2 | $CD11b^+F4/80^+CD206^+CD86^-$ |
| Monocyte | $CD115^+CD11b^+Ly\text{-}6G^-$ |
| Neutrophil | $Ly\text{-}6G^+CD11b^+Gr1^+Ly\text{-}6C^-Siglec\text{-}F^-CD19^-$ |
| Eosinophil | $Siglec\text{-}F^+CD11b^+Ly6C^{low}Ly\text{-}6G^-$ |
| Basophil | $Fc\varepsilon RI\alpha^+CD49b^+CD11b^+Ly6C^{low}CD3^-CD19^-Ly\text{-}6G^-Siglec\text{-}F^-$ |

Th, T helper cell; $T_{fh}$, follicular helper T cell; Treg, regulatory T cell; $CD4^+$ T Naïve, naïve $CD4^+$ T cell; $CD4^+$ $T_{CM}$, central memory $CD4^+$ T cell; $CD4^+$ $T_{EM}$, effector memory $CD4^+$ T cell; $CD8^+$ T Naïve, naïve $CD8^+$ T cell; $CD8^+$ $T_{CM}$, central memory $CD8^+$ T cell; $CD8^+$ $T_{EM}$, effector memory $CD8^+$ T cell; NK T, natural killer T cell, DC, dendritic cell; cDC, conventional dendritic cell; pDC, plasmacytoid dendritic cell; NK cell, natural killer cell; M1, M1 macrophage; M2, M2 macrophage.
