# Supplementary material for: Thymic stromal lymphopoietin promotes abdominal aortic aneurysm formation by regulating macrophage polarization
Source: Front Immunol. 2026 Apr 7;17:1767913. doi: 10.3389/fimmu.2026.1767913 (PMC13095562; doi:10.3389/fimmu.2026.1767913)
Supplement: Supplementary file 2 [file DataSheet2.pdf]

frontiers

The International Society for the Advancement of Cytometry (ISAC) has highlighted the importance of including comprehensive methodological information to ensure data reproducibility and reliability. In line with this, Frontiers in Immunology now requires authors to submit a checklist for manuscripts that involve flow or mass cytometry. This checklist helps standardize the reporting process, improving the quality and transparency of published data. By doing so, we support scientific progress, making it easier for other researchers to replicate and validate experiments.

This form should be submitted with any manuscripts using flow or mass cytometry.

Sample/specimen/material description

☐ Total blood

☑ PBMCs

☑ Organ digests

Other mouse aorta

Did the samples suffer any treatment before or after incubation with the antibodies?

☑ Drug thymic stromal lymphopoietin

☑ Cell permeabilization #00552300, eBioscience, USA

☐ Dye ______

☐ Propidium iodine

☐ Not applicable

Other ______

Instrument and antibodies

Name of the Cytometer Helios2 Mass Cytometer ; DxFLEX

Antibodies and targets	Fluorochrome/ Metal	Catalog number/Company
e.g. anti-CD4	FITC	Cat. XXX/ XXX ltd.
anti- CD86	FITC	105005/ Biolegend

anti- F4/80	BV421	123137/ Biolegend
anti- CD206	PerCP/Cyanine5.5	141716/ Biolegend
anti- CD45	89Y	PLT TECH
anti- CD3e	115In	PLT TECH
anti- Ki67	139La	PLT TECH
anti- Gr1	141Pr	PLT TECH
anti- TCRgd	142Nd	PLT TECH
anti- CD69	143Nd	PLT TECH
anti- CXCR3	144Nd	PLT TECH
anti- XCR1	145Nd	PLT TECH
anti- Ly6G	147Sm	PLT TECH
anti- Ly6C	148Nd	PLT TECH
anti- CD117	149Sm	PLT TECH
anti- CD44	150Nd	PLT TECH
anti- B220	151Eu	PLT TECH
anti- CD11c	152Sm	PLT TECH
anti- CD19	153Eu	PLT TECH
anti- CD83	154Sm	PLT TECH
anti- CCR7	155Gd	PLT TECH
anti- Bcl6	156Gd	PLT TECH
anti- FceRIa	157Gd	PLT TECH
anti- IgM	158Gd	PLT TECH
anti- F4/80	159Tb	PLT TECH
anti- CD62L	160Gd	PLT TECH
anti- CD25	162Dy	PLT TECH
anti- Tim4	163Dy	PLT TECH
anti- CD86	164Dy	PLT TECH
anti- FOXP3	165Ho	PLT TECH

anti- CCR4	166Er	PLT TECH
anti- CD206	167Er	PLT TECH
anti- CXCR5	168Er	PLT TECH
anti- CD317	169Tm	PLT TECH
anti- CD161	170Er	PLT TECH
anti- TSLP	171Yb	PLT TECH
anti- CCR2	172Yb	PLT TECH
anti- CCR6	173Yb	PLT TECH
anti- TCRb	174Yb	PLT TECH
anti- SiglecF	175Lu	PLT TECH
anti- MHCII	176Yb	PLT TECH
anti- CD4	197Au	PLT TECH
anti- CD8a	198Pt	PLT TECH
anti- CD11b	209Bi	PLT TECH

Data analyses

1. Name of the software FlowJo™ Software
2. Reference gating strategy in the manuscript or supplementary material

Gating strategy in (eg Figure X) Figure 5F, Supplementary Figure 2; Supplementary Table 2
